# β1 adrenoceptor antibodies induce myocardial apoptosis via inhibiting PGC-1α-related pathway

**DOI:** 10.1186/s12872-020-01492-3

**Published:** 2020-06-05

**Authors:** Linying Shi, Jia Liu, Yuan Zhang, Mulei Chen, Jiamei Liu

**Affiliations:** Heart Failure Center, Department of Cardiology, Beijing Chao-Yang Hospital, Capital Medical University, 8 Gongrentiyuchangnan Rd, Beijing, 100020 China

**Keywords:** Peripartum cardiomyopathy, Autoantibody against β1 adrenoceptor, Peroxisome proliferator-activated receptor γ coactivator-1α, Vascular endothelial growth factor, Apoptosis

## Abstract

**Background:**

Peripartum cardiomyopathy (PPCM) is life-threatening heart disease. However, the causes and pathogenesis of PPCM remain unclear. Previous studies found that β1 adrenoceptor antibodies (β1AA) had possible involvement in the development of PPCM. In the present study, we determined the potential relationship between PPCM and β1AA, including the mechanism of β1AA leading to PPCM.

**Methods:**

We extracted the β1AA from the postpartum Wistar rats that were injected by the antigen peptide segment of the β1 adrenoceptor to produce PPCM. We tested the effects of β1AA on H9C2 cell line by CCK-8, LDH, TUNEL, SA-ELISA, qRT-PCR, and western blot methods. Furthermore, PGC-1α was overexpressed to rescue the effect of β1AA on H9C2 cells.

**Results:**

We found that the extracted β1AA induced apoptosis of cardiac myocytes of H9C2 cell line. Moreover, the expression of peroxisome proliferator-activated receptor γ coactivator-1α (PGC-1α), which is a master regulator of mitochondrial metabolism, and its downstream transcript vascular endothelial growth factor (VEGF) got decreased in H9C2 cells after β1AA treatment. In addition, the effect of β1AA could be inhibited by atenolol, the antagonist of β1 adrenoceptors (β1AR) and imitated by isoprenaline, the agonist of β1AR. Furthermore, overexpression of PGC-1α in the H9C2 cells rescued the apoptosis of cells and inhibitory expression of VEGF induced by β1AA.

**Conclusions:**

Our results suggest that the symptoms of PPCM due to myocardial cell apoptosis induced by β1AA inhibiting the PGC-1α-related pathway impairs mitochondrial energy metabolism. Therefore, our results uncover a previously unknown role of the β1AA pathway in the etiology of PPCM and provide a novel potential target for the treatment of PPCM.

## Background

β1 adrenoceptor (β1AR) plays an important role in adrenergic regulation of myocardial contractility, which induces positive chronotropic, inotropic, and dromotropic action on myocardial through stimulatory Gs protein [1]. However, from the past two decades, researchers found that the β1-adrenoceptor antibodies (β1AA) participate in the dilated cardiomyopathy (DCM). Peripartum cardiomyopathy (PPCM) is one kind of DCM. The pregnant women who are in good health seem to have cardiac failure for unknown reasons [2]. Although patients with PPCM have no prior history of heart disease and there are no other known possible causes of heart failure, our previous study found that there was a high correlation between β1AA and the development of PPCM [3]. However, the mechanisms involved in β1AA inducing PPCM are not fully understood.

PPCM, defined as idiopathic cardiomyopathy, represents heart failure secondary to left ventricular systolic dysfunction towards the end of pregnancy or in the months following delivery [4]. It was found that inappropriate signaling and metabolic derangement at the level of the mitochondrion might be one of the key reason leading to myocardial hypertrophy [5, 6]. In addition, mitochondria play a major role in apoptosis, which is greatly associated with heart failure [7]. Reports state that β1AA induce apoptosis in cardio myocytes isolated from normal rats [8], suggesting the mechanism of PPCM induced by β1AA may relate to mitochondrial dysfunction.

Peroxisome proliferator-activated receptor γ coactivator-1α (PGC-1α) is a key integrator of transcriptional circuits regulating mitochondrial biogenesis and function [9]. Activation of the PGC-1α regulatory cascade increases cardiac mitochondrial oxidative capacity in the heart [9] and protects the cells from apoptosis [10]. To test the hypothesis that β1AA inducing PPCM relates to mitochondrial dysfunction, initially, we developed immune PPCM models by injecting the antigenic peptide segment of β1AR into postpartum Wistar rats and extracted the autoimmune β1AA from these animals. Later, we examined the effects of extractive β1AA on survival and apoptosis in myocardial H9C2 cells and the expression of β1AR, caspase3, PGC-1α, and vascular endothelial growth factor (VEGF), which is the downstream transcript of PGC-1α. Finally, we overexpressed the PGC-1α in H9C2 cells to observe whether it can rescue the cell’s apoptosis after β1AA treatment.

## Methods

### Study design

Previous study found that there was a high correlation between β1AA and development of PPCM [3], indicating a possible involvement of β1AA in the development of PPCM. In the present study, β1AA were extracted from the postpartum Wistar rats, which were injected the antigen peptide segment of the β1 adrenoceptor to produce autoimmunity and the effects of extracted β1AA on the H9C2 cells were examined.

### Animals and active immunity

Ten postpartum Wistar rats were obtained from Model Animal Research Center of Nanjing University, maintained in specific pathogen-free (SPF) conditions under a 12 h-light-12 h-dark cycle. All animal experiments were approved by the Institutional Animal Care and Use Committee (IACUC) of Nanjing University, Nanjing, China, and was conducted in accordance with the Guide for the Care and Use of Laboratory Animals from the National Institutes of Health (Bethesda, MD, USA). Animals were randomly divided into two groups, and five in each group. The antigenic peptide segment of β1 adrenoceptor was purchased from GL Biochem company (Shanghai, China). For first immunization, the antigenic peptide segment was dissolved into the Na_2_CO_3_ solution, and mixed the solution with freund’s incomplete adjuvant 1: 1. The final concentration of antigenic peptide segment in the mixture was 1 mg/ml. Then the mixture was injected subcutaneously in back of each animal with antigenic peptide segment 0.4 μg/g. For the negative control, Na_2_CO_3_ solution was injected with the same protocol. Two weeks after the first immunization, immune process was repeated again. Intravenous blood samples were performed on each groups in the third week. After animal experiments, the animals were euthanized with intraperitoneal injection of excessive pentobarbital (100-150 mg/kg).

### Autoimmune β1AA procedure extraction

The affinity chromatography method was employed to extract proteins from serum. Four grams of the Sepharose4B (Sigma, Aldrich, USA) were immobilised onto 30 ml Sepharose column according to the manufacturer’s instructions (Amersham, Uppsala, Sweden). Three millilitres of conditioned or control serum were 1:8 diluted in PBS respectively, and applied onto the coupled-column at room temperature. After extensive washing with PBS until the value of OD280 < 0.05, the bound proteins were eluted with 3 M MgCl_2_. The eluted proteins were collected for each IgG according to the value of OD280 and dialyzed against PBS. The eluted proteins were analyzed by 12% SDS/PAGE under reducing conditions. In addition, quantification of the eluted proteins was determined by BCA (bicinchonininc acid) assay.

### Cells culture and transfection experiments

H9C2 cells were purchased from cell bank of the Chinese Academy of Sciences (Shanghai, China, Cat#: CNR 5) and cultured in F12-DMEM medium with 10% fetal bovine serum. All these cells were incubated at 37 °C, 5% CO_2_. The medium was changed for 2 days and the cells were subcultured. One day before experiments, the cultured cells were digested with 0.25% trypsin + 0.02% EDTA. After digestion, cells were triturated to free collected by centrifugation (1500 rpm, 5 min). Then, cells were seeded in a 96-well plate at a density of 1 × 10^4^ cells per well and incubated at 37 °C, 5% CO_2_ over night. After these treatments, 1 μM β1AA, 1 μM nonspecific IgG, 1 μM isoprenaline or mixture of 1 μM β1AA and 1 μM atenolol were added into the cell-culture medium with processing 12 h. Then, subsequent experiments were carried out. For overexpression of PGC-1α, the expression plasmid pcDNA3-PGC-1α encoding PGC-1α was constructed by our laboratory and plasmid expressing shRNA for PGC-1α and its control shRNA was purchased from Thermo Scientific. Cells were transiently transfected with plasmid for 24 h before the experiments, using Lipofectamine® 2000 Transfection Reagent (Invitrogen, Carlsbad, American, Cat#: 11668–019) according to the manufacturer’s recommendations.

### CCK-8 assay

Cytotoxicity of β1AA was evaluated by CCK-8 assay in H9C2 cells. The CCK-8 detection kit was purchased from 7sea Biotech company (Shanghai, China, Cat#: 20140419). Briefly, after treatments, a 10-ul of CCK-8 or F12-DMEM medium were added to each well and the cells were further incubated for 1 h and the absorbance of each well was measured using a Microplate Reader (Model 680, BIO-RAD, USA) at the wavelength of 450 nm.

### LDH assay

Measurements of LDH release were performed following the manufacturer’s instructions. The LDH detection kit was purchased from Promega company (Madison, American, Cat#: G7891) After cells were exposed to β1AA or nonspecific IgG suspensions, the exposure medium was collected and centrifuged at 1500 rpm for 10 min. The supernatant was mixed with the LDH assay mixture at a ratio of 1:1 and incubated at room temperature in the dark for 30 min. The reaction was stopped by the addition of 1 N HCl (1/10 of mixture volume), and the absorbance at 590 nm. Viability in relation to the control (the unexposed group) is calculated from equation:

Viability(%Control) = (LDH_lysed_ − LDH_Exposed_) / (LDH_lysed_ − LDH_control_)(100%).

where LDH_lysed_ is the LDH released from wells treated with the LDH lysing solution (total cellular LDH content), LDHexposed is the LDH released from wells exposed to β1AA or nonspecific IgG suspensions, and LDH control is the LDH released from cells in the control group.

### Terminal deoxynucleotidyltransferase-mediated dUTP nick-end labeling (TUNEL) of myocardial cells

A fluorometric TUNEL detection kit (Genecopoeia, Rockerville, USA, Cat#: A050) was used to detect apoptotic DNA strand breaks. The H9C2 cells were fixed with 4% neutral buffered formaldehyde in PBS (pH 7.4) at 25 °C for 30 min, permeated with 50 μg/ml proteinase K at 25 °C for 15 min, and incubated with the labeling reaction mixture in a humidified chamber at 37 °C for 1 h. The cells were then processed with a standard immunocytochemical staining procedure to incubate with antibody against DAPI (a cell nucleus marker; Invitrogen, Carlsbad, CA). Finally, a leica fluorescence microscope (DM6000B, Leica, Germany) was used to capture the images, and the ratio of TUNEL positive nuclei in total (DAPI positive nuclei) was computed to express the cells apoptosis.

### Sa-ELISA

To test the concentration of VEGF, SA- ELISA was performed. Precisely, the medium of cell cultures was added into a 96-well plate 100 μl per well. The wells were then incubated for 1 h at 37 °C with antibody labeled with biotin anti-rat monoclonal IgG (1:1000, 50 μL/well) (BOSTER, Cat#: BA1005). Next, streptomycin with horseradish peroxidase (1:500, 50 μL/well) (Bioss, Cat#: bs-0437P-HRP) was added into the wells and incubated for 1 h at 37 °C. Finally, chromogenic tetramethylbenzidine (TMB) solution (50 μL/well) was added into each well and incubated for 10-30 min at 37 °C. Optical density (OD) was measured at 450 nm on a micro plate reader (Erba Lisa Scan II, Germany). The OD value was compared with the standard and the final concentration was obtained.

### Quantitative real time-PCR (qRT-PCR)

Total RNA of H9C2 cells was isolated using Ultrapure RNA Kit (Cat#: CW0581S, CWBio). 400 ng of RNA was subjected to reverse transcription-PCR with SuperRT cDNA Synthesis Kit (Cat#: CW0741S, CWBio) according to the instruction. Quantitative RT-PCR was performed with PCR primers listed in Table 1. UltraSYBR Mixture Kit (Cat#: CW2602M, CWBio) was employed to detect mRNA levels of these genes. All reactions were repeated 3 times and GAPDH was used to normalize target.

**Table 1:** List of utilized primers for qRT-PCR

Gene	Forward primer	Reverse primer
β1AR	CGACTGCTGGTGCTCGCGTCG	AGCGAAAGGGCAGCGTGATGGC
PGC-1α	CGCACAACTCAGCAAGTCCTC	CCTTGCTGGCCTCCAAAGTCTC
PGC-1β	CAAGAAGCGGCGGGAAA	GCTCATGTCACCGGAGAGATTT
VEGF	GCAGCGACAAGGCAGACTATT	ACCGTTGGCACGATTTAAGAG
NRF1	CCACGTTGGATGAGTACACG	CTGAGCCTGGGTCATTTTGT
ERRα	AAGCCCTGATGGACACCTC	GAAGCCTGGGATGCTCTTG
GAPDH	TGGAGTCTACTGGCGTCTT	TGTCATATTTCTCGTGGTTCA

### Western blot analysis

The H9C2 cells were lysed in RIPA buffer (P0013C, Beyondtime) containing 1 mM PMSF (ST505, Beyondtime). Total protein of 30 μg was subjected to electrophorese on 12–6% SDS-Page gels and transferred to PVDF membranes. Antibodies against β1AR (1:1000; cat. bs-0498R, Bioss), VEGF (1:800; cat. AF5131, Affinity), PGC-1α (1:1000, cat. bs-1832R, Bioss), and caspase3 (1:1000; cat. bs-0081R, Bioss) were used as primary antibodies. Rabbit IgG antibodies coupled to horseradish peroxidase (HRP) were used as secondary antibodies. GAPDH (1:1000; cat. BA2913, Boster) was used as loading control. An enhanced chemiluminescence (ECL) system was used for detection of protein bands.

### Statistical analysis

All data were presented as mean ± SEM. One-way ANOVA was conducted to evaluate the one-way layout data. If a significant difference was observed, Bonferroni’s post-hoc test was conducted to identify groups with significant differences. The relative mRNA levels were calculated using the 2-ΔΔCt method. All analyses were performed using SPSS 19.0. Differences were considered significant with *p* < 0.05.

## Results

### Validation of extractive β1AA

**Fig. 1:**
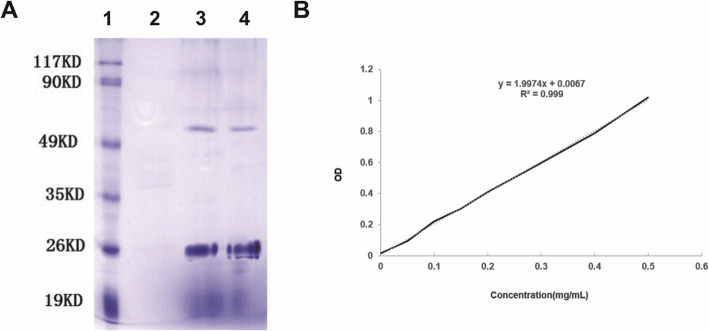
Validation of extractive β1AA. **a** SDS-PAGE gel electrophoresis raw data of serum. Lane 1, Marker; Lane 2, Negative serum; Lane 3, Serum of autoimmune postpartum animals; Lane 4, Standard sample. **b** Standard curve of OD-concentration. The protein concentration of Serum of autoimmune postpartum animals was 3.63 mg/kg

To investigate the effect of β1AA on the PPCM first, β1AA were extracted from the autoimmune postpartum Wistar rats by using the Affinity chromatography method. In Fig. 1a, the negative sera lacked bands whereas the immune group had two bands in the sera at 55 KD and 25 KD, which remained consistent with the standard products and we also tested the concentration of the β1AA in the serum where the final concentration was 3.63 mg/ml (Fig. 1b).

### The effect of extractive β1AA on survival and apoptosis in myocardial H9C2 cells

**Fig. 2:**
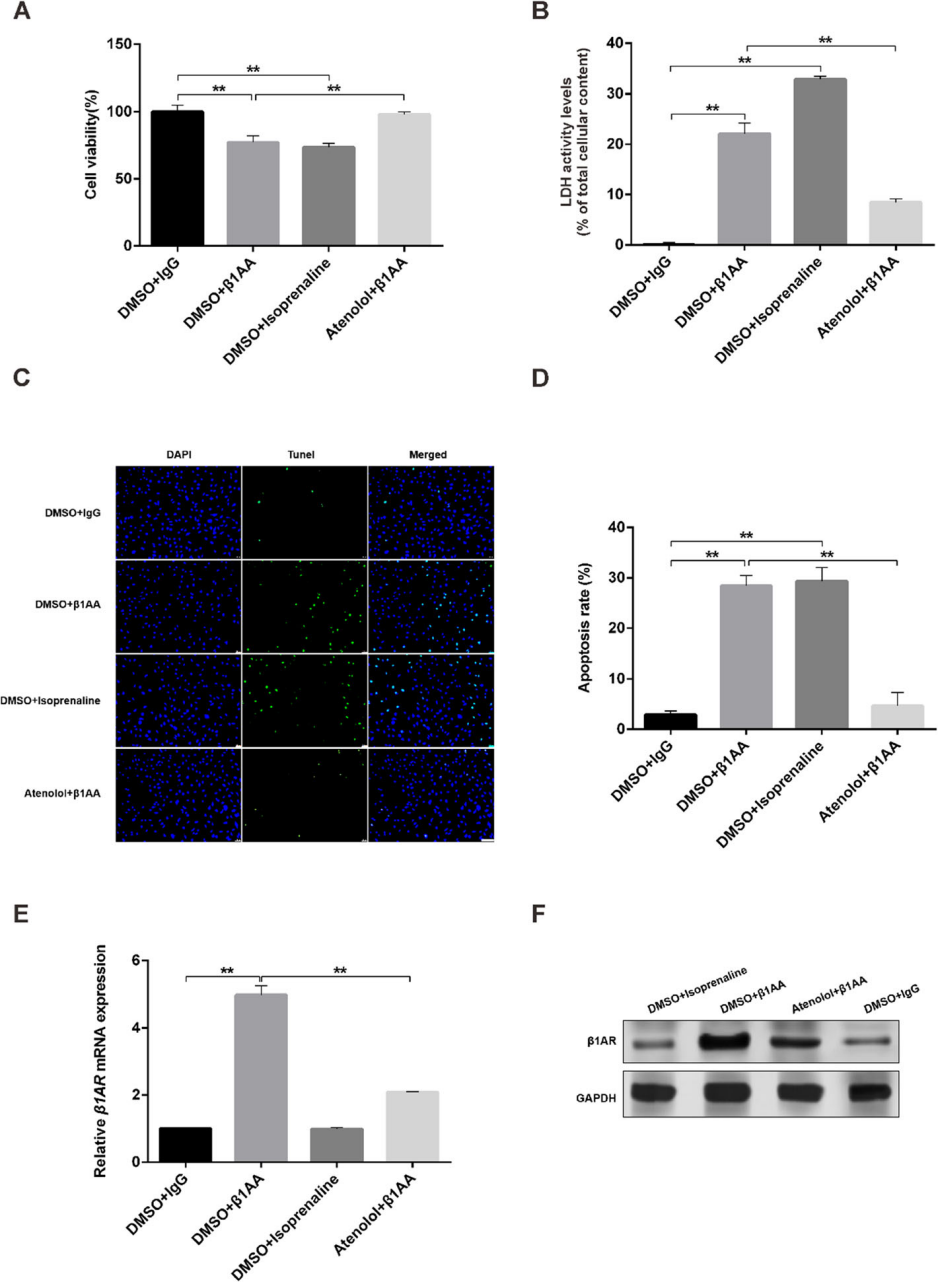
Extractive β1AA decreased the rate of survival and increased the rate of apoptosis in myocardial H9C2 cells. **a** The results of CCK8 assay. β1AA significantly inhibited the cell proliferation. The effect of β1AA could be mimiced by isoprenaline, an agonist of β1AR and be inhibited by atenolol, an antagonist of β1AR. **b** The results of LDH assay. β1AA significantly increased the apoptosis of H9C2 cells. This effect could also be mimicked by isoprenaline and inhibited by atenolol. **c** TUNEL positive nuclei obtained from H9C2 cells visualized by fluorescence microscopy. Scale bar, 100 μm. **d** Group data of apoptosis rate of TUNEL assay (*n* = 5). E and F. β1AA significantly inreased the mRNA and protein expression of the β1AR (n = 5). The effects of β1AA on β1AR could be inhibited by atenolol. **, *P* ≤ 0.01. Group data presented by mean ± SEM

The results of the CCK8 assay (Fig. 2a) indicated that β1AA significantly inhibited cell proliferation compared with the control group. From the previous studies, we found that β1AA was a weak agonist of the β1AR; hence we used isoprenaline, the agonist of the β1AR and atenolol, the antagonist of β1AR to mimic and inhibit the effect of β1AA on inhibitory proliferation in H9C2 cells. We found that isoprenaline had the same effect as β1AA and atenolol could reverse the inhibitory effect on the proliferation of H9C2. We found similar results in LDH (Fig. 2b) and Tunel (Fig. 2c and d) assay. The apoptosis of H9C2 cells significantly increases after β1AA and isoprenaline treatment. And atenolol inhibited the effect of β1AA on apoptosis of H9C2 cells. Finally, compared to the negative IgG group, the mRNA and protein level of β1AR significantly increased after β1AA treatment and atenolol inhibited the effect of β1AA on the expression (Fig. 2e and f). However, although isoprenaline was the agonist of β1AR as β1AA, it did not affect the expression of β1AR. All above results suggested that the effect of β1AA on H9C2 cells was through β1AR.

### β1AA inhibited the PGC-1α related factor expression

**Fig. 3:**
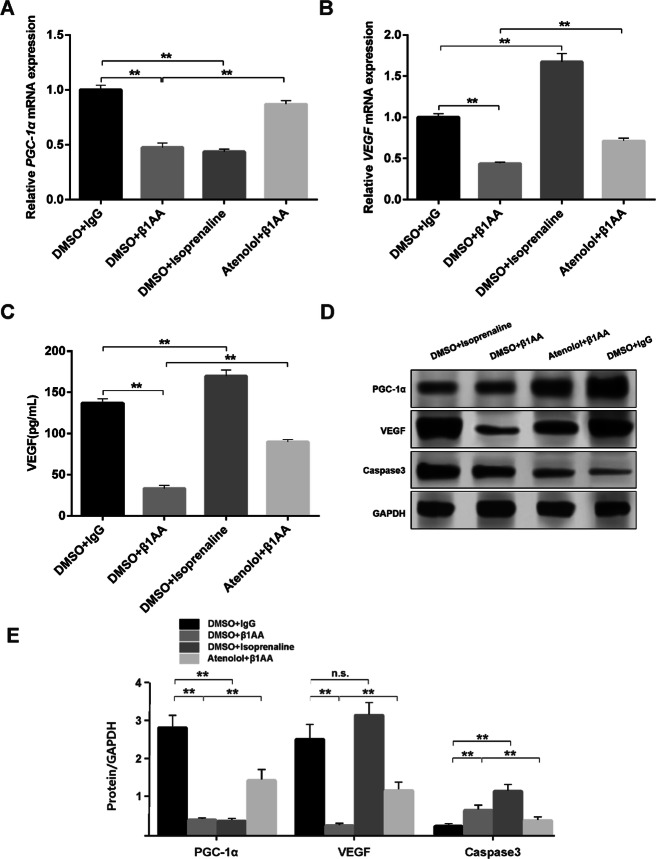
β1AA inhibited the PGC-1α related factor expression. β1AA significantly inhibited the mRNA expression of PGC-1α (**a**) and VEGF (**b**). And the effect of β1AA on mRNA expression of PGC-1α could also be mimicked by isoprenaline. However, isoprenaline promoted the mRNA expression of VEGF (n = 5). **c***ELISA* test for secreted VEGF in the supernatant of the medium (n = 5). **d** Raw data of results of WB test. **e** Relative expression content of PGC-1α, VEGF and caspase3 (n = 5). β1AA increased the protein expression of caspase3, and inhibited the protein expression of PGC-1α and VEGF. The effects of β1AA could be inhibited by atenolol. And the effect of β1AA on protein expression of PGC-1α could also be mimicked by isoprenaline. However, isoprenaline promoted the protein expression of VEGF expression rather than inhibition as β1AA did. **, *P* ≤ 0.01. n.s., no statistical significance. Group data presented by mean ± SEM

We examined the PGC-1α related factor expression after β1AA treatment. Both β1AA and isoprenaline, inhibited the expression of PGC-1α mRNA and atenolol inhibited the effect of β1AA (Fig. 3a). Furthermore, β1AA inhibited the expression of VEGF mRNA, while isoprenaline promoted its expression. Atenolol also inhibited the effect of β1AA on the expression of VEGF mRNA (Fig. 3b). The results of the ELISA test for secreted VEGF in the supernatant of the medium show that β1AA inhibited VEGF secretion and atenolol inhibited the effect of β1AA (Fig. 3c). Isoprenaline promoted the secretion of VEGF, which was the same as mRNA results (Fig. 3c). In addition, as shown in Fig. 3d and e, the expression of caspase3 protein increased obviously, while the expression of PGC-1α and VEGF protein decreased after β1AA treatment. Atenolol can inhibit these effects. Although isoprenaline inhibited the expression of PGC-1α protein and activated the expression of caspase3 protein like β1AA, whereas increased the expression of VEGF, which is contrary to β1AA. Altogether, these results suggested that β1AA could inhibit the PGC-1α related factor expression.

### Overexpression of PGC-1α rescued β1AA induced apoptosis of H9C2 cells

**Fig. 4:**
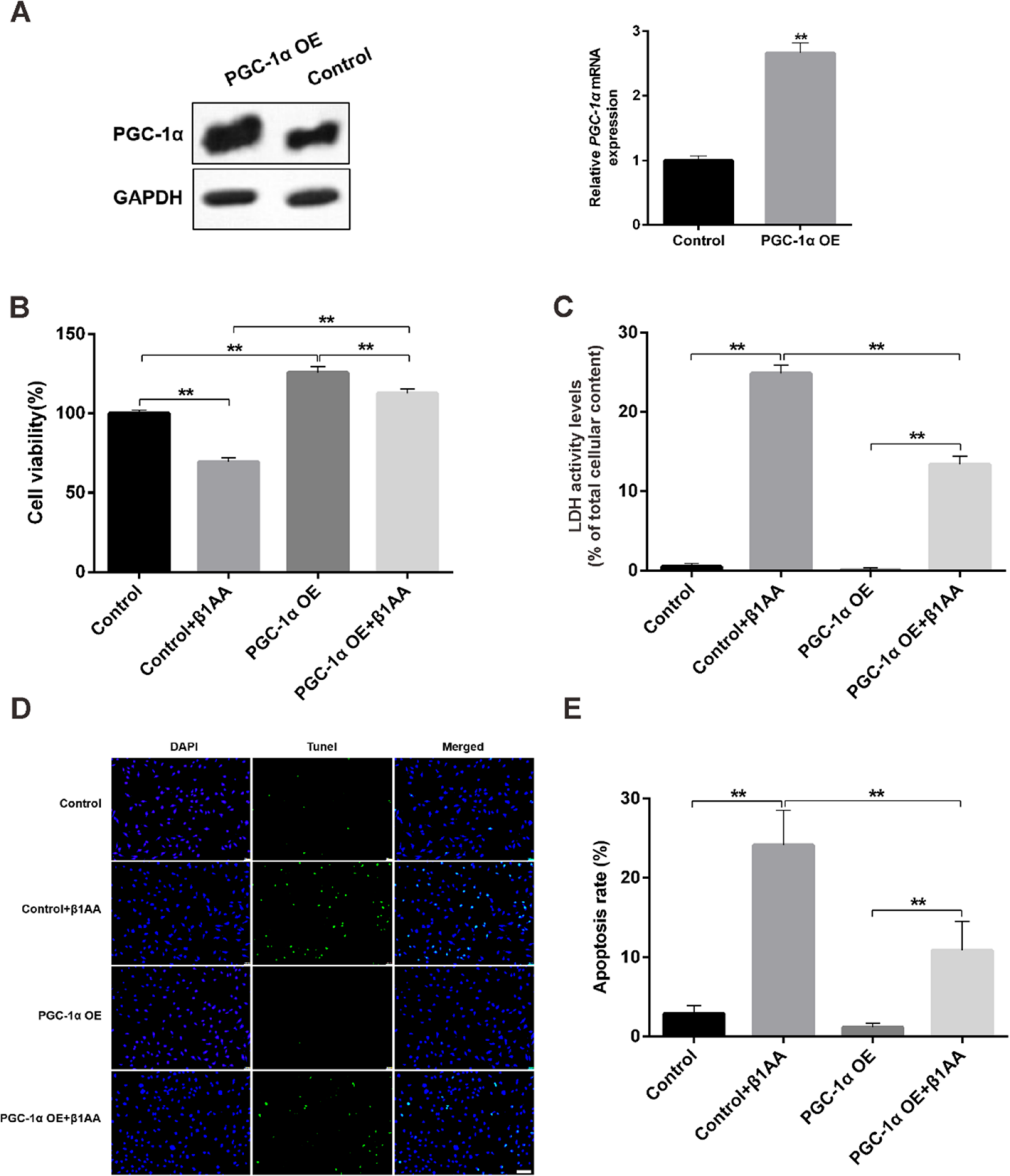
Overexpression of PGC-1α rescued β1AA induced apoptosis of H9C2 cells. **a** The expression of protein and mRNA of PGC-1α in the PGC-1α overexpression H9C2 cells. **b** Overexpression of PGC-1α rescued the inhibition of β1AA to cell proliferation in CCK8 assay. **c** Overexpression of PGC-1α decreased the apoptosis of the cells and inhibited the role of β1AA in promoting apoptosis in LDH assay. **d** TUNEL positive nuclei obtained from H9C2 cells visualized by fluorescence microscopy. Scale bar, 100 μm. **e** Group data of apoptosis rate of TUNEL assay. **, P ≤ 0.01. Group data presented by mean ± SEM

Next, we overexpressed PGC-1α in H9C2 cells. The expression of mRNA and protein of PGC-1α had a significant increase in the overexpressed H9C2 cells (Fig. 4a). It suggested the successful build-up of the cell models. Then we tested the cell proliferation by CCK8 assay and found that β1AA inhibited cell proliferation compared to the control group in the cells transfusing empty plasmids. However, overexpression of PGC-1α significantly rescued the inhibition of β1AA to cell proliferation (Fig. 4b). We obtained similar results in the LDH (Fig. 4c) and Tunel assay (Fig. 4d and e). The apoptosis of H9C2 cells significantly increased after β1AA treatment. But overexpression of PGC-1α significantly decreased the apoptosis rate of the cells and inhibited the role of β1AA in promoting apoptosis.

### Overexpression of PGC-1α rescued the inhibitory effect of β1AA on VEGF expression

**Fig. 5:**
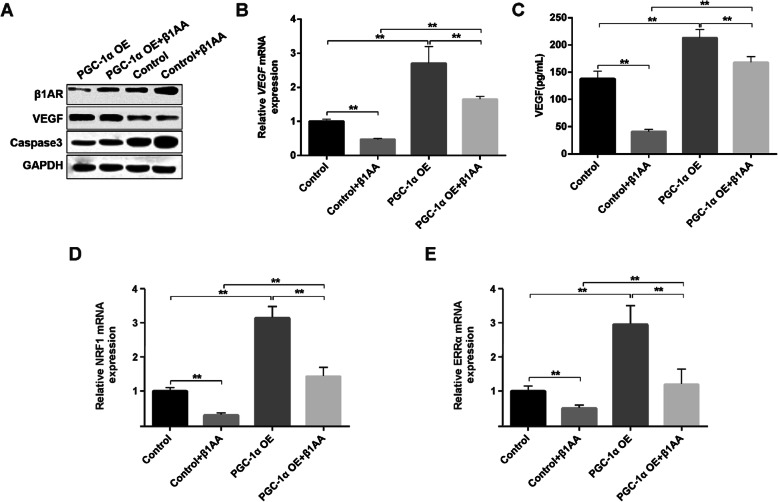
Overexpression of PGC-1α rescued the inhibitory effect of β1AA on VEGF expression. **a** Raw data of results of WB test. β1AA increased the protein expression of β1AR and caspase3, and inhibited the protein expression of VEGF. But the overexpression of PGC-1α inhibited the effect of β1AA. **b** Overexpression of PGC-1α increased the VEGF mRNA expression and rescued the inhibitory effect of β1AA. **c** ELISA test for secreted VEGF in the supernatant of the medium. Overexpression of PGC-1α also rescued the inhibitory effect of β1AA on the VEGF secretion. **d** and **e** overexpression of PGC-1α increased the NRF1 and ERRα mRNA expression and rescued the inhibitory effect of β1AA. **, P ≤ 0.01. Group data presented by mean ± SEM

The expression of β1AR and caspase3 protein increased and, the expression of VEGF protein decreased after β1AA treatment in the cells transfusing empty plasmids (Fig. 5a and b). In comparison to cells transfected with empty plasmids, cells with overexpressed PGC-1α had lower expression of β1AR, caspase 3 proteins, and increased expression of VEGF protein, even after β1AA treatment. We also tested the expression of VEGF mRNA in each group. The results showed that the β1AA significantly inhibited the expression of VEGF mRNA, and the overexpression of PGC-1α rescued the inhibitory effect of β1AA on expression of VEGF mRNA (Fig. 5c). We found similar results in ELISA test for secreted VEGF in the supernatant of the medium. β1AA significantly inhibited the VEGF secretion, and PGC-1α overexpression rescued the inhibitory effect of β1AA on secretion of VEGF. Because it has been reported that PGC-1α could regulate mitochondrial biogenesis and function [11, 12]. Finally, we tested the mRNA expression of NRF1 and ERRα, which were associated with mitochondrial biogenesis. Quantitative PCR analyses showed specific down-regulation of NRF1 and ERRα after β1AA treatment and it was rescued by overexpression of PGC-1α. All these results suggest that β1AA inducing myocardial cell apoptosis is by inhibiting the PGC-1α pathway, which leads to disorder of cell mitochondrial metabolism.

## Discussion

The Heart Failure Association of the European Society of Cardiology Working Group defined PPCM as idiopathic cardiomyopathy presenting with heart failure secondary to left ventricular systolic dysfunction towards the end of pregnancy or in the months following delivery, where no other cause of heart failure is found [4]. PPCM occurs in 1/1000–1/4000 pregnancies, more commonly in women of African ancestry [13]. Perhaps it is found up to 1% of all pregnancies in countries like Haiti and Nigeria [14, 15]. Only 25% of PPCM patients in developing countries survive up to 5 years, with associated infant mortality of 50–75% [16]. Our results showed that β1AA, which were extracted from postpartum Wistar rats that were injected by the antigen peptide segment of the β1 adrenoceptor, could induce apoptosis of cardiac myocytes of H9C2 cell line and the effect of β1AA on H9C2 cells might be caused by inhibition of PGC-1α related pathway.

Although the pathophysiological mechanism is not known, abnormal autoimmunity response to pregnancy and β1AA are associated with high risk for the progression and prevalence of PPCM. Because the serum level of β1AA in patients with DCM is higher than in normal people [17, 18] and later the immunization of animals with a synthetic peptide corresponding to the second extracellular loop of β1AR leads to the production of IgG autoantibodies against this domain [19]. After several months of immunization, the animals showed left ventricular hypertrophy and contractile dysfunction [20]. β-blockers have been used successfully for decades to treat several pathologies, including hypertension, congestive heart failure, and post-MI dysfunction [21]. Moreover, chronic β-blocker treatment could alter baseline leukocyte characteristics that decrease their responsiveness to acute injury, and it means prior β-blockade may act to reduce the severity of innate immune responses [22]. Since β1AA is a weak agonist of β1AR, its accumulation might lead to the opposite effect of the blockers. Finally, as an adrenoceptor ligand, the sirloin protein fully involves in cardiac inflammatory, oxidative, and apoptotic processes [23, 24]. Although in diabetic patients, the protective anti-apoptotic and anti-remodeling effect played by drugs on the cardiovascular system not implies in the regulation of sympathetic tone and β-adrenoreceptors [25, 26], our previous study found that there was a high correlation between β1AA and development of PPCM [3]. This difference may be due to the effects of different diseases on cardiomyocytes. Most recently, experimental progress has strongly suggested a causal role for hormonal insults in PPCM [2]. However, there is no direct evidence for β1AA induced PPCM. Here, we first proved that β1AA, which were extracted from the postpartum rats with autoimmunity, directly induce apoptosis in H9C2 cardiac myocytes through β1AR. It signifies that apoptosis plays a pathophysiological role in heart failure [27]. Thus, these results suggest that β1AA may directly contribute to the pathogenesis of PPCM.

Our data show that β1AA inhibited the PGC-1α related factor expression and VEGF secretion. Moreover, PGC-1α overexpression rescued apoptosis of cardiac myocytes induced by β1AA and the inhibitory effect of β1AA on PGC-1α related factor expression and VEGF secretion. It suggests that the mechanism of β1AA induced apoptosis is through inhibiting the PGC-1α related pathway. PGC-1α plays a critical role in the augmentation of mitochondrial biogenesis, cellular respiration rates, and energy substrate uptake and utilization [10]. Down regulation of the PGC-1α has been described both in patients with nonischemic cardiomyopathy and in animal models of pressure overload and heart failure [28–30]. Furthermore, in the PGC-1α-KO models, PGC-1α deletion resulted in reduced palmitate oxidation and increased glucose oxidation. PGC-1α-KO hearts exhibited impaired inotropic and chronotropic response [31–33]. In addition, in present study, β1AA showed specific down-regulation of genes associated with mitochondrial biogenesis (NRF1 and ERRα). Therefore, the inhibitory effect of β1AA on PGC-1α related factor expression might cause mitochondria dysfunction, later leading to heart failure. On the other hand, under the ischemia condition, PGC-1α expression can induce skeletal muscle cells leading to VEGF secretion to regenerate the blood vessels [34]. Recently, heart failure remains associated with microvascular sparsity [35]. Our data shows β1AA inhibition of VEGF secretion, leading to a decrease in microvascular density, suggesting another reason for β1AA induced PPCM. The diagnosis of PPCM is difficult to make, as it is a diagnosis of exclusion, with a considerable overlap with other conditions. Especially in women who present with acute heart failure at the end of pregnancy or directly postpartum, thorough investigations and intensive follow-up may often lead to alternative diagnoses [4]. Our findings suggest the increase of β1AA together with the decrease of PGC-1α as a diagnostic tool for PPCM. However, more clinical studies are needed to prove this deduction.

β1AA are the weak agonist of β1AR. We also found atenolol, the antagonist of β1AR can inhibit the effect of β1AA, suggesting the function of β1AA, realized through β1AR. However, isoprenaline is the non-selective agonist of β adrenoceptor, it cloud active both β1AR and β2 adrenoceptors (β2AR) [1]. The difference between effects of β1AA and isoprenaline on the expression of β1AR might be caused by mechanism of function, including receptor affinity and dynamic properties of the β-agonist. Activation of β2AR has been reported to promote VEGF secretion [36] and restore mitochondrial function of kidney [37] and podocyte [38] through PGC-1α dependent mitochondrial biogenesis. It seems that the activation of β2AR would lead to increase of VEGF and PGC-1α expression. We thought the different effects of isoprenaline on PGC-1α and VEGF are the result of the integration of its activation of β1AR and β2AR at the same time. However, the effects of activations of different β adrenergic receptor on expression of β adrenergic receptor and PGC-1α related pathway should be further studied.

## Conclusions

In conclusion, our present findings establish that β1AA directly induce apoptosis in H9C2 cardiac myocytes through β1AR. The reduced expression of PGC-1α and VEGF, as well as the consequently elevated apoptosis rate of myocytes, may account for the β1AA-induced PPCM. Therefore, our results uncover a previously unknown role of the β1AA pathway in the etiology of PPCM,and provide a novel potential target for the treatment of PPCM.

## Data Availability

The dataset used and/ or analyzed during the current study are available from the corresponding author on reasonable request.

## Abbreviations

β1AR: β1 adrenoceptors
β2AR: β2 adrenoceptors
β1AA: β1 adrenoceptor antibodies
DCM: Dilated cardiomyopathy
PPCM: Peripartum cardiomyopathy
PGC-1α: Peroxisome proliferator-activated receptor γ coactivator-1α
VEGF: Vascular endothelial growth factor
